# Elucidation of the Binding Mechanism of Coumarin Derivatives with Human Serum Albumin

**DOI:** 10.1371/journal.pone.0063805

**Published:** 2013-05-28

**Authors:** Archit Garg, Darla Mark Manidhar, Mahesh Gokara, Chandramouli Malleda, Cirandur Suresh Reddy, Rajagopal Subramanyam

**Affiliations:** 1 Department of Plant Sciences, School of Life Sciences, University of Hyderabad, Hyderabad, India; 2 Department of Chemistry, Sri Venkateswara University, Tirupathi, Andhrapradesh, India; 3 Department of Biochemistry, School of Life Sciences, University of Hyderabad, Hyderabad, India; Medical School of Hannover, United States of America

## Abstract

Coumarin is a benzopyrone which is widely used as an anti-coagulant, anti-oxidant, anti-cancer and also to cure arthritis, herpes, asthma and inflammation. Here, we studied the binding of synthesized coumarin derivatives with human serum albumin (HSA) at physiological pH 7.2 by using fluorescence spectroscopy, circular dichroism spectroscopy, molecular docking and molecular dynamics simulation studies. By addition of coumarin derivatives to HSA the maximum fluorescence intensity was reduced due to quenching of intrinsic fluorescence upon binding of coumarin derivatives to HSA. The binding constant and free energy were found to be 1.957±0.01×10^5^ M^−1^, −7.175 Kcal M^−1^ for coumarin derivative (CD) enamide; 0.837±0.01×10^5^ M^−1^, −6.685 Kcal M^−1^ for coumarin derivative (CD) enoate, and 0.606±0.01×10^5^ M^−1^, −6.49 Kcal M^−1^ for coumarin derivative methylprop (CDM) enamide. The CD spectroscopy showed that the protein secondary structure was partially unfolded upon binding of coumarin derivatives. Further, the molecular docking studies showed that coumarin derivatives were binding to HSA at sub-domain IB with the hydrophobic interactions and also with hydrogen bond interactions. Additionally, the molecular dynamics simulations studies contributed in understanding the stability of protein-drug complex system in the aqueous solution and the conformational changes in HSA upon binding of coumarin derivatives. This study will provide insights into designing of the new inspired coumarin derivatives as therapeutic agents against many life threatening diseases.

## Introduction

Coumarin is a naturally occurring benzopyrone found in variety of plants. Coumarin and its derivatives have roles as anti-inflammatory, anti-coagulant, anti-retroviral, anti-arthritic, anti-herpes, anti-asthmatic, and antioxidant activities [1]–[7].Their anti-retroviral activity is directly linked to their inhibitory effect on HIV-1 replication. The reduction in reverse transcriptase activity was observed when HIV infected ACH-2 lymphocytes were treated with warfarin, 4-hydroxycoumarin and umbelliferone [1]. They were used as the anti-inflammatory agents as they inhibit the cyclooxygenase and 5-lipoxygenase activities which convert arachidonic acid to endoperoxides, precursors of prostaglandins and to leukotriene A4, respectively. Inhibition of prostaglandin synthesis accounts for their analgesic and antipyretic actions and inhibition of leukotriene accounts for their use in the treatment of asthma and mild arthritis [2]. The anti-herpes simplex virus activity was identified in 7-(carboxymethoxy)-4-methyl coumarin designed by virtual combinatorial synthesis and selected by computational screening [3]. It has also been used in the treatment of lymphedema [4]. It also exhibits anticoagulant property when converted to dicoumarin by the strain of fungi *Aspergillus fumigatus* [5]. Further, it inhibits the release of plasma clotting factor VII by vitamin K without inhibition of protein synthesis [6]. Umbelliferone acts as a scavenger of reactive oxygen species which accounts for its antioxidant property [7].

In order to understand the pharmacokinetics and pharmacodynamics of the drugs, study of interaction between drugs and the plasma proteins is essential. Human serum albumin (HSA), the most abundant protein in the blood plasma, tends to have high affinity for variety of metabolites and drugs. HSA is used as a carrier to deliver various exogenous and endogenous substances including hormones, fatty acids and drugs to the target organs [8]–[18]. HSA is synthesized and secreted from liver cells. It is a 67 KDa protein having single chained non glycosylated polypeptide which folds into a heart shaped protein with approximately 67% α-helical content [17]–[19].The globular protein of HSA comprises of three structurally similar α-helical domains (I–III) each consisting of subdomains (A and B), and stabilized by 17 disulphide bridges. Aromatic and heterocyclic ligands have been found to bind mainly within subdomains IIA and IIIA designated as site I and site II, respectively [19]–[25]. Also, seven binding sites are localized in sub-domains IB, IIIA and IIIB, and on the sub-domain interfaces [18], [23], [26]. Many endogenous substances and drugs such as bilirubin, hemin, azapropazone, indomethacin, and tri-iodobenzoic acid (TIB) have been found to bind within IB domain and substances such as diflunisal, halothane, and ibuprofen bind to IIA–IIB domain [25]. HSA contains a single intrinsic tryptophan residue at position 214 in domain IIA where its fluorescence is sensitive to the closely associated ligands [27], [28]. Thus, fluorescence measurement is used as a probe for the drug binding studies with HSA and also used for differentiation of various fatty acid compositions in oils [29]. Recently our group has shown that natural compounds like maslinic acid, trimethoxy-flavone, coumaroyltyramine, β-sitosterol, and betulinic acid binds strongly to HSA leading to a change in protein conformation [30]–[35].

**Figure 1:**
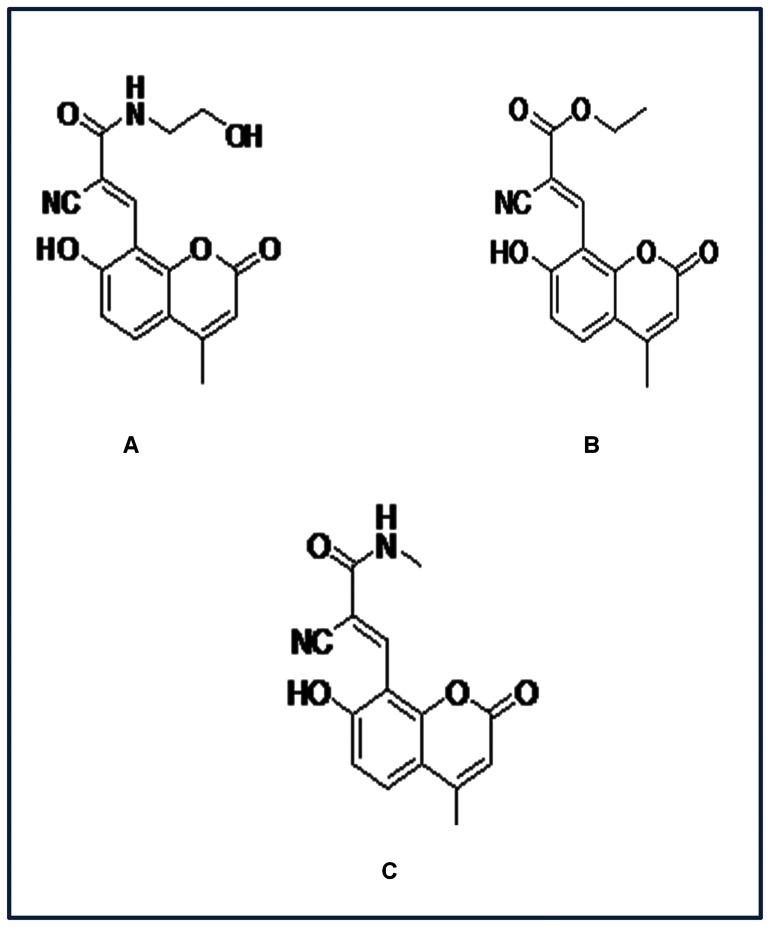
Chemical structures of 7-hydroxy-4-methyl coumarin derivatives with their IUPAC names. A) (2E)-2-cyano**-**N-(2-hydroxyethyl)-3-(7-hydroxy-4-methyl-2-oxo-2H-chromen-8-yl) prop-2enamide (CD enamide). Molecular mass-314.29 Da. Molecular formula- C_16_H_14_N_2_O_5_. B) Ethyl (2E)-2-cyano-3-(7-hydroxy-4-methyl-2-oxo-2H-chromen-8-yl) prop-2-enoate (CD enoate). Molecular mass- 299.28 Da. Molecular formula- C_16_H_13_NO_5_. C) (2E)-2-cyano-3-(7-hydroxy-4-methyl-2-oxo-2H-chromen-8-yl)-N-methylprop-2-enamide (CDM enamide). Molecular mass- 284.27 Da. Molecular formula- C_15_H_12_N_2_O_4_.

The coumarin derivatives which have been synthesized (IUPAC names) are, 1) (2E)-2-cyano**-**N-(2-hydroxyethyl)-3-(7-hydroxy-4-methyl-2-oxo-2H-chromen-8-yl)prop-2-enamide [Coumarin derivative (CD) enamide], 2) Ethyl (2E)-2-cyano-3-(7-hydroxy-4-methyl-2-oxo-2H-chromen-8-yl) prop-2-enoate [Coumarin derivative (CD) enoate], and 3) (2E)-2-cyano-3-(7-hydroxy-4-methyl-2-oxo-2H-chromen-8-yl)-N-methylprop-2-enamide [Coumarin derivative methylprop (CDM) enamide] (Figure 1). A comparative study of interactions of these coumarin derivatives with major carrier protein HSA is important as these are supposed to have several beneficial drug properties like coumarin. Hence, here we studied the molecular interactions of HSA with the coumarin derivatives under physiological conditions (pH 7.2) using fluorescence spectroscopy, circular dichroism (CD) spectroscopy, molecular docking studies and molecular dynamics (MD) simulations.

## Materials and Methods

### Synthesis of Coumarin Derivatives

7-hydroxy-4-methyl coumarin was synthesized by reacting resorcinol and ethylacetoacetate in presence of concentrated sulfuric acid under *Pechmann’s conditions* which we have published recently [36]. This compound was formylated with hexamethylenetetramine (HMTA) and acetic acid *under Duff’s conditions* to get 7-hydroxy-4-methyl-8-formyl coumarin (I).

The compound(I) was under *Knoevengel’s conditions* treated with N-substituted cyanoacetamide with catalytic amount of piperidine in ethanol. The substituted cyanoacetamide derivatives were prepared by reacting ethylcyanoacetate and an amine. The compound identification was done by nuclear magnetic resonance (NMR). See the brief scheme of the synthesis (Figure S1).

### Preparation of Stock Solutions

Fat-free HSA was dissolved in physiological aqueous solution of 100 mM phosphate buffer, pH 7.2 at a concentration of 2.9 mM. The optimum physiological pH for HSA was set to 7.2 as it has the maximum absorption at this pH [30]. Coumarin derivatives were prepared (1 mM) in 20∶80% ethanol:water mixture. A solution containing 20% ethanol has no effect on secondary structure of HSA [30], [31]. We also examined the maximum time taken by the coumarin derivatives in binding to HSA by fluorescence emission and CD spectroscopy and found that 10 min is the maximum binding time and hence, 10 min incubation time was used for all the experiments. All the chemicals were purchased from Sigma Aldrich.

### Fluorescence Spectroscopy

The fluorescence emission spectra were recorded on a Perkin Elmer LS55 Fluorescence spectrometer from 300 to 450 nm, with an excitation wavelength of 285 nm. The slit width for excitation and emission was 5 nm. The final concentration of coumarin derivatives were 0.001, 0.002, 0.003, 0.004, 0.005, 0.006, 0.007, 0.008, 0.009 and 0.01 mM in 100 mM phosphate buffer pH 7.2 with the fixed concentration of HSA 0.001 mM. The binding constant and free energy were calculated using the maximum fluorescence value at 362 nm. Three independent experiments were performed, and identical spectra were obtained.

### Circular Dichroism Spectroscopy

CD spectra of HSA-coumarin derivatives were recorded with JASCO 810 spectropolarimeter, using a quartz cell of path length 0.2 cm. Three scans were accumulated with continuous scan mode and a scan speed of 50 nm min^−1^with data being collected at every nm from 190 to 300 nm. The final concentration of HSA was fixed to 0.001 mM and the concentrations of coumarin derivatives used were 0.001, 0.003 and 0.005 mM in 100 mM phosphate buffer pH 7.2. The protein secondary structure was calculated using CDNN 2.1 software.

### Molecular Modeling and Docking

Molecular docking is the computational process of searching for a ligand that is able to fit both geometrically and energetically the binding site of a protein. Docking was done using AutoDock 4.2.3 [37]. It uses the Genetic Algorithm (GA) for internal conformation search and generates an ensemble of conformations by implementing Lamarckian Genetic Algorithm [38].

#### Genetic algorithm

Genetic Algorithm is a computer program that mimics the process of evolution by structures called chromosomes. Each of these encodes a possible solution (in terms of a possible ligand-receptor interaction) to the docking problem and may be assigned a fitness score on the basis of the relative merit of that solution. Each chromosome encodes an internal conformation and protein active site and includes a mapping from hydrogen bonding sites in the ligand and protein. On decoding a chromosome, a least-squares fitting process is employed to position the ligand within the active site of the protein. The fitness of a decoded chromosome is then a combination of the number and strength of the hydrogen bonds that have been formed in this way and of the van der Waals energy of the bound complex [39], [40].

#### Preparation of protein and ligand

The known crystal structure of HSA (PDB ID: 1AO6) was obtained from the Brookhaven Protein Data Bank [22]. From the 2D structure, the three-dimensional structure of coumarin derivatives was built, and the geometry was optimized through the Gabedit 2.2.12 software [41]. In AutoDock, water molecules and ions were removed (including ordered water molecules) and hydrogen atoms added at appropriate geometry groups within the protein, were ionized as required at physiological pH. The structure of HSA was protonated in AutoDock 4.2.3.

#### Genetic Algorithm parameters used

The parameters used for genetic algorithm were number of GA runs:30; Population size:150; Maximum number of energy evaluations:2500000; Maximum number of generations:27000; Maximum number of top individuals that automatically survive :1; Rate of genetic mutation:0.02; Rate of crossover:0.8; GA crossover mode: two points; Mean of Cauchy distribution for gene mutation: 0.0; Variance of Cauchy distribution for gene mutation: 1.0; Number of generations for picking worst individual: 10. Thirty genetic algorithm runs were performed without early termination. Output was selected as Lamarckian GA(4.2) and file was saved as.dpf.

#### AutoDock Analysis

AutoDock generated the different ligand conformers using a Lamarkian GA, a GA implementation with an adaptive local method search [38]. The simulations started with a predefined number of generation cycles, composed of mapping and fitness evaluation, selection, crossover, mutation and elitist selection steps, and continued with a local search, followed by the scoring of the generated conformers. The energy-based AutoDock scoring function includes terms accounting for short range van der Waals and electrostatic interactions, loss of entropy upon ligand binding, hydrogen bonding, and solvation. The protein and the ligand input structures, prepared as described above, were transformed into the corresponding.pdbqt format output files (containing atom coordinates, partial charges, and salvation parameters). To recognize the binding sites in HSA, blind docking was carried out, the grid size set to 126, 126, and 126 along the *X*, *Y*, and *Z* axes with 0.697 Å grid spacing. The center of the grid set to 29.5, 31.8, and 23.5 Å. During docking, a maximum number of 30 conformers was considered, and the root mean square (rms) cluster tolerance was set to 2.0 Å. One of the lowest energy conformations was used for further analysis which matches the experimental values.

### Molecular Dynamics Simulations

Molecular dynamics (MD) simulations are the important tools used for the theoretical study of biological molecules. These are used for understanding the physical basis of structure and function of macromolecules [42]. MD simulations provide the details about time-dependent behavior of a molecular system. These permit the study of complex dynamic processes that occur in the biological systems such as the effect of solvent on protein structure and stability [43], [44], transport of ligand and ions in biological systems [45] and also provide the means to determine the NMR structure [46] and carry out the refinement of X-ray structure [47]. Here in this study, a 10,000 ps MD simulation of the complex was carried out with the GROMACS4.0 [48], [49] package using the GROMOS96 43a1 force field [50], [51]. The initial conformation was taken from one of the lowest binding energy docking conformation (Table S1). The topology parameters of HSA were created by using the Gromacs program. The topology parameters of coumarin derivatives were built by the Dundee PRODRG2.5 server (beta) [52]. Then the complex was immersed in a cubic box (7.335×6.135×8.119 nm^3^) of extended simple point charge (SPC) water molecules [50]. The solvated system was neutralized by adding sodium ions in the simulation, and the entire system was composed of 5843 atoms of HSA, one coumarin derivative, 15 Na^+^ counter ions and 69491 solvent atoms. To release conflicting contacts, energy minimization was performed using the steepest descent method of 1000 steps, followed by the conjugate gradient method for 1000 steps. MD simulation studies consist of equilibration and production phases. In the first stage of equilibration, the solute (protein, counter ion, and coumarin derivative) was fixed and the position-restrained dynamics simulation of the system, in which the atom positions of HSA were restrained at 300 K for 30 ps. Finally, the full system was subjected to 10,000 ps MD at 300 K temperature and 1 bar pressure. The periodic boundary condition was used and the motion equations were integrated by applying the leaf-frog algorithm with a time step of 2 fs. The atom coordinates were recorded every 0.5 ps during the simulation for latter analysis.

## Results and Discussion

### Identification of Binding Affinity from Fluorescence Emission Spectra

**Figure 2:**
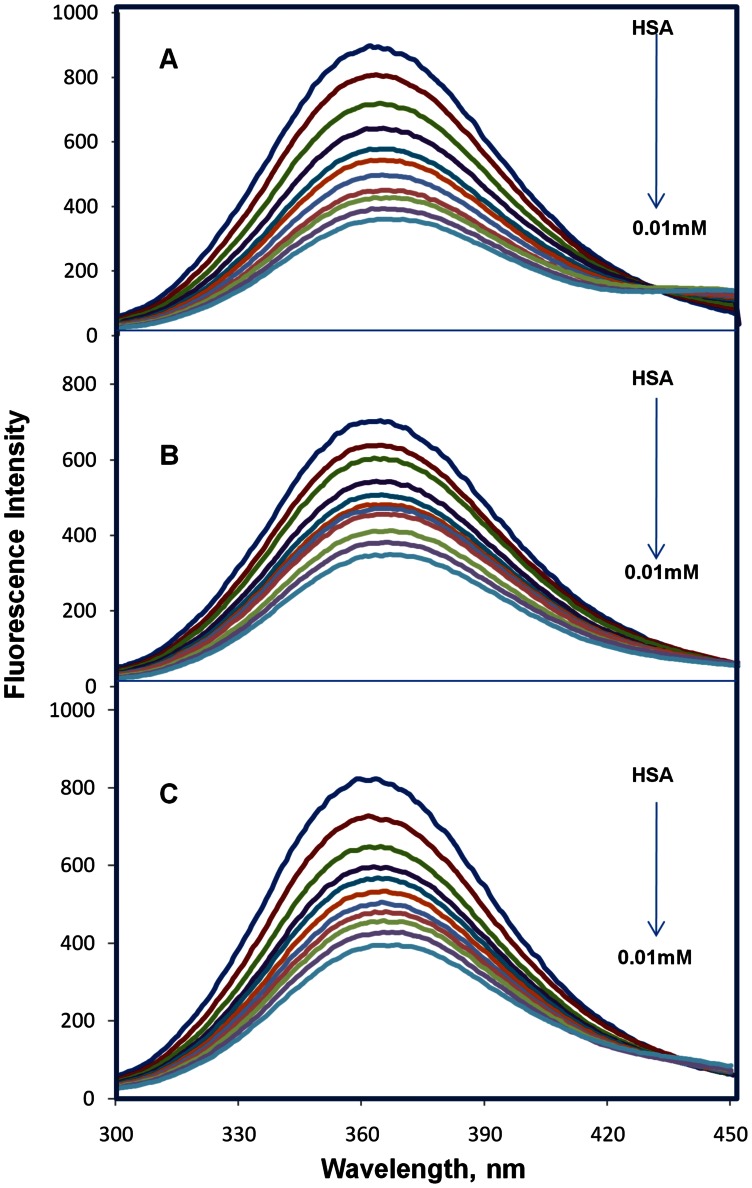
Fluorescence emission spectra of HSA-coumarin derivatives in 100 mM phosphate buffer pH 7.2, λ_ex_ = 285 nm. (A) free HSA (0.001 mM) and free HSA with different concentrations of CD enamide (0.001-0.01 mM). (B) free HSA (0.001 mM) and free HSA with different concentrations of CD enoate (0.001-0.01 mM). (C) free HSA (0.001 mM) and free HSA with different concentrations of CDM enamide (0.001-0.01 mM).

Fluorescence quenching of the protein upon drug binding was used to find the drug-protein interactions and measure the binding affinity of the drug to HSA [53]. Figure 2 depicts the room temperature fluorescence emission spectra of HSA obtained fluorescence maximum at 362 nm, which is in agreement with our previous reports [30], [31]. In the presence of different concentrations of coumarin derivatives (0.001 to 0.01 mM) with the physiological phosphate buffer pH 7.2, our results showed that, with the increasing concentrations of coumarin derivatives and a fixed concentration of HSA (0.001 mM), the maximum fluorescence (362 nm) of HSA was quenched upon binding of coumarin derivatives (Figure 2). However, there is no peak shift at the maximum fluorescence upon binding of coumarin derivatives. This indicates that binding of coumarin derivatives to HSA causes microenvironment changes in HSA and leads to HSA-coumarin derivatives complexes. The decrease in fluorescence intensity is known as quenching, which may be due to the interaction of the excited state of fluorophore with its surroundings [54]. The maximum fluorescence emission of HSA comes from tryptophan, tyrosine, and phenylalanine. Phenylalanine has a very low quantum yield and the fluorescence of tyrosine is almost totally quenched if it is ionized or present near to an amino group, a carboxyl group or a tryptophan. Thus, the fluorescence of HSA is dominated by the tryptophan emission, and the emission spectrum of HSA is mainly from a single residue of Trp-214 in subdomain IIA. A change in the intrinsic fluorescence intensity of HSA was due to the tryptophan residue when small molecules bind to HSA [55]. Similar fluorescence quenching results were reported for several ligands [32]–[34], [56], [57], [58]. Our group has recently reported the similar quenching results for coumaryltyramine, betulinic acid, feruloyl masalinic acid, trimethoxy flavone and β-sitosterol and also HSA-drug complexes were formed [30]–[34].

**Figure 3:**
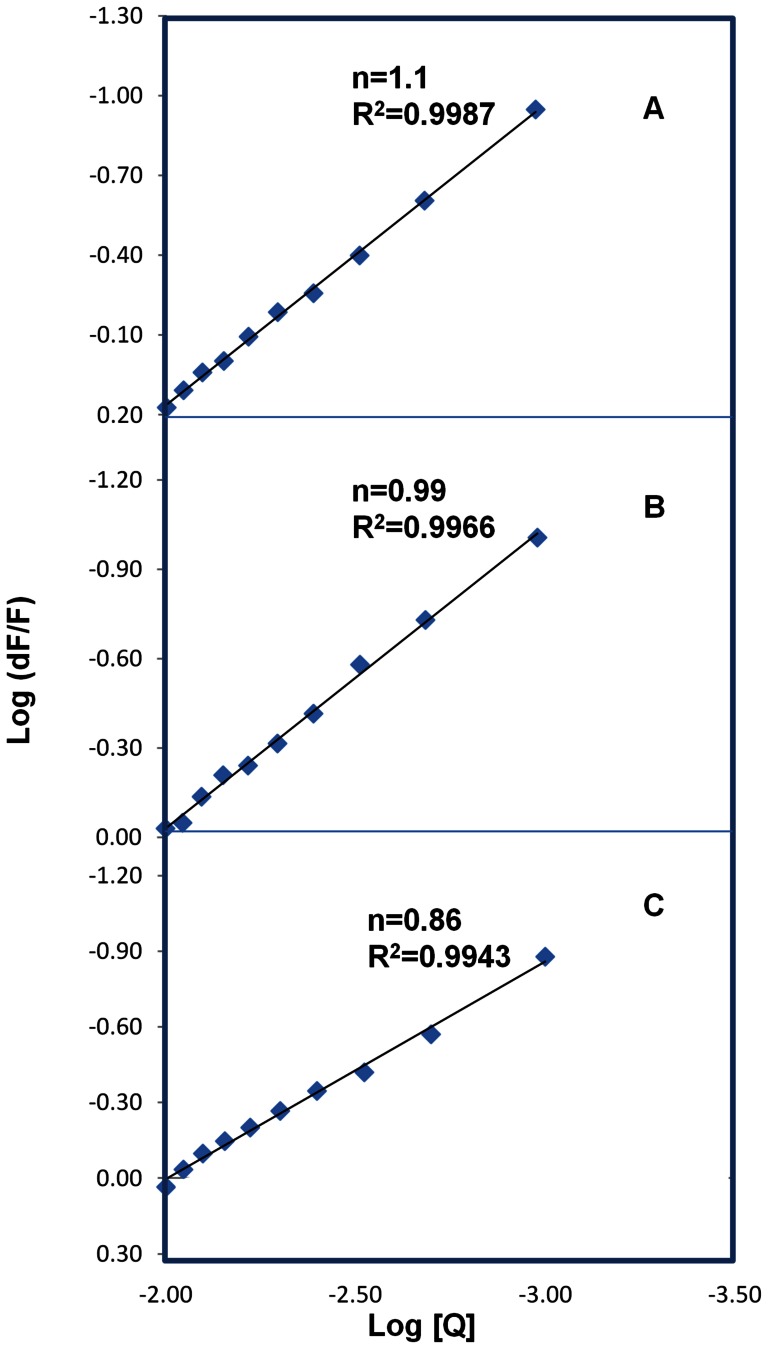
The Modified Stern−Volmer plots. (A) plot of log (d*F*/*F*) against log [*Q*] for CD enamide. (B) plot of log (dF/F) against log [Q] for CD enoate. (C) plot of log (dF/F) against log [Q] for CDM enamide. *λ*_ex_ = 285 nm, *λ*_em_ = 362 nm.

The binding constant (*K*_s_) and the number of binding sites (*n*) can be derived from the modified Stern-Volmer plot according to the following equation [59]

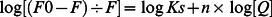
(1)where *n* is the number of ligands binding to the protein, *K_s_* is the binding constant, [*Q*] is the drug concentration, F_0_ is the initial fluorescence of free HSA and F is fluorescence with different concentrations of coumarin derivatives. The result indicated a good linear relationship. The number of binding ligands were calculated to be 1.1, 0.99 and 0.86 for CD enamide, CD enoate and CDM enamide respectively (Figure 3), suggesting that HSA interacts with different coumarin derivatives in a one-to-one ratio. The binding constants K_CD enamide_, K_CD enoate_ and K_CDM enamide_ were calculated from the intercept as 1.957±0.01×10^5^, 0.837±0.01×10^5^ and 0.606±0.01×10^5^ M^−1^, respectively which indicates strong binding of these derivatives to HSA which is in agreement with the previously reported interaction of different coumarin derivatives with HSA [58]. It is also in good correlation with the computationally calculated binding constant as 9.5×10^4^, 7.95±0.01×10^4^ and 4.65×10^4^ M^−1^, respectively for K_CD enamide_, K_CD enoate_ and K_CDM enamide_ where they have lowest free energy (Table S1). Thus, the above results are in agreement with our previous reports of, betulinic acid, feruloyl maslinic acid, trimethoxy flavones, coumaroyltyramine and β-sitosterol were showing binding constants with HSA of *K*_BA_ = (1.685±0.01)×10^6^ M^−1^, *K*_FMA_ = (1.42±0.01)×10^8^ M^−1^, K_TMF_ = (1.0±0.01)×10^3^ M^−1^, K_CT_ = (4.5±0.01)×10^5^ M^−1^, K_βS_ = (4.6±0.01)×10^3^ M^−1^ [30]–[34].

### Free Energy Calculation from the Binding Constant

The standard free energy change can be calculated from the equation:
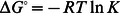
(2)where ΔG° is the free energy change, R is the gas constant and K is the binding constant at the room temperature (which can be obtained from the fluorescence data).It is known that binding constant obtained from fluorescence emission can be used to calculate the free energy and also different binding interactions like hydrogen bonds, hydrophobic, van der Waals forces, and electrostatic interactions. Thus, the free energy change calculated upon binding of CD enamide, CD enoate and CDM enamide to HSA were −7.175, −6.685 and −6.49 Kcal/mol, respectively at 25°C. This indicates that the free energy of binding for these complexes derives mainly from hydrophobic and possibly hydrogen bond interactions. Interestingly, the computationally calculated free energy is also closely matching to the experimental values which are −6.8, −6.69 and 6.37 Kcal/mol for CD enamide, CD enoate and CDM enamide upon binding to HSA at 25°C (Table S1). Similar types of interactions were observed from our recent studies of natural compounds, feruloyl maslinic acid, trimethoxy flavone, coumaroyltyramine and β-sitosterol with HSA [31]–[34].

### Conformational Changes Observation Obtained from Circular Dichroism Spectroscopy

**Figure 4:**
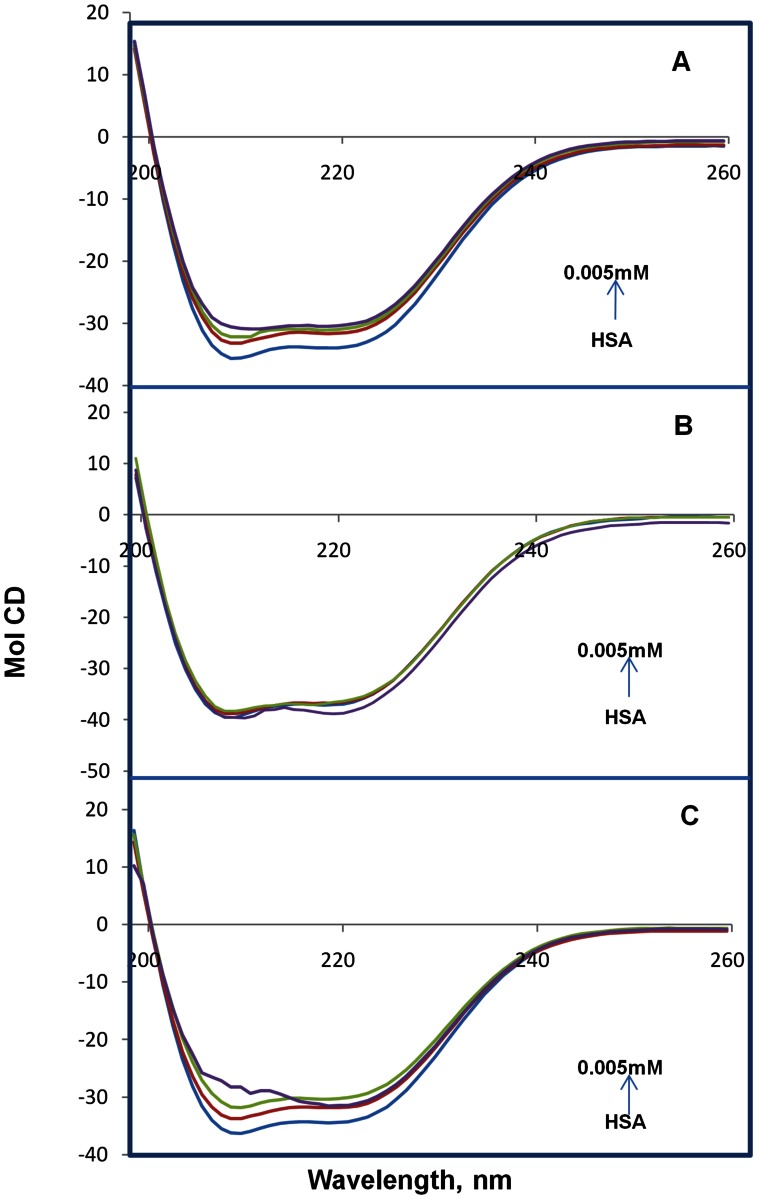
Circular dichroism of the free HSA and HSA+coumarin derivatives complexes. (A) the free HSA and HSA+CD enamide complexes in aqueous solution with a HSA concentration of 0.001 mM and CD enamide concentrations were 0.001, 0.003 and 0.005 mM. (B) the free HSA and HSA+CD enoate complexes in aqueous solution with a HSA concentration of 0.001 mM and CD enoate concentrations were 0.001, 0.003 and 0.005 mM. (C) the free HSA and HSA+CDM enamide complexes in aqueous solution with a HSA concentration of 0.001 mM and CDM enamide concentrations were 0.001, 0.003 and 0.005 mM. All experiments were repeated five times and obtained identical spectra.

**Figure 5:**
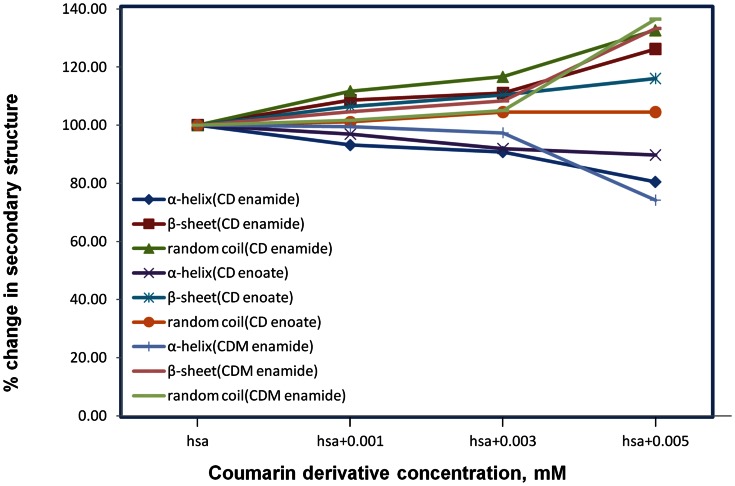
Secondary structure composition calculated from **Figure 4** **.** Concentration dependent secondary structure change for free HSA, HSA-CD enamide complex, HSA-CD enoate complex and HSA-CDM enamide complex.

CD spectroscopy is a sensitive technique to obtain the secondary structural conformational changes in the protein. The CD spectra of HSA exhibit two negative bands at 208 and 218 nm. The CD spectra of HSA exhibited two negative bands in the UV region at around 208 and 218 nm, which are characteristic of the α-helix of protein, and both contributed to n-π* transfer for the peptide bond of the α-helical structure [30]–[34]. The secondary structure was determined using CDNN 2.1 software. When various concentrations of different coumarin derivatives were added to free HSA and incubated for 10 min, the intensities at 208 and 218 nm decreased in a concentration-dependent manner (Figure 4). This clearly demonstrates considerable changes in the protein secondary structure especially the α-helix structure. In free HSA, the secondary protein conformation found to be ∼57.3% α-helix, ∼24.9% β-sheet (including parallel and anti-parallel) and 17.8% random coil which is in close agreement with our previous reports [30]–[34]. From our results, it was found that upon complexation of HSA with different coumarin derivatives (0.001, 0.003 and 0.005 mM), the α-helical content decreased from 57 to 47, 53 and 43% with a increase in β-sheets from 25 to 31, 29 and 33% and random coils from 18 to 22, 19 and 24% for CD enamide, CD enoate and CDM enamide, respectively (Table S2, Figure 5).The proportion of secondary structural elements undergoes marginal variation at low concentrations of coumarin derivatives (Figure 5). However, at the higher concentrations of coumarin derivatives, there was a noticeable change in the secondary structure of protein. These results suggest that the secondary structure of HSA became partially unfolded due to HSA-coumarin derivatives complexes formation. Secondary structural conformational changes occur as interior flexibility of the IB–IIA domain of HSA due to binding of different coumarin derivatives which have been shown from docking studies. Recently, our group reported that *N*-*trans*-*p*-coumaroyltyramine, a natural compound bound to HSA, leads to conformational changes due to inherent flexibility at the IIA domain [33]. Similar results were observed upon binding of other ligands (pentacyclic triterpenoids derivatives of betulinic acid and feruloyl maslinic acid, trimethoxy flavone, coumaroyltyramine and β-sitosterol) to HSA, thus indicating a major decrease of α- helices and an increase of β-sheets as well as random coils [30]–[34].

### Molecular Docking Studies

**Figure 6:**
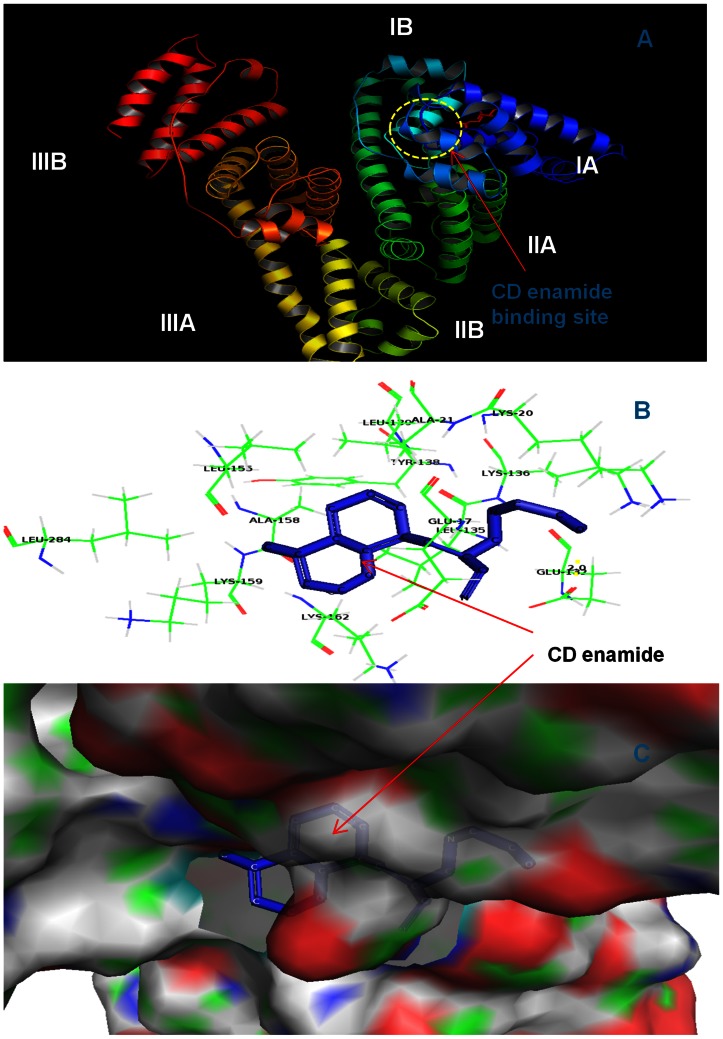
CD enamide docked in the binding pocket of HSA using Autodock4.2. **Different view of HSA and CD enamide docked conformation.** (A) overview in cartoon model of CD enamide binding to HSA, (B) the docking poses of the HSA-CD enamide complex depicted in a ball and stick model (blue), and HSA, represented in line model, (C) hydrophobic pocket of HSA and CD enamide, the CD enamide represents in ball and stick model (blue) and the binding pockets in cyanocolor, in which the green color represents the binding pocket residues. Images are generated using PyMol.

**Figure 7:**
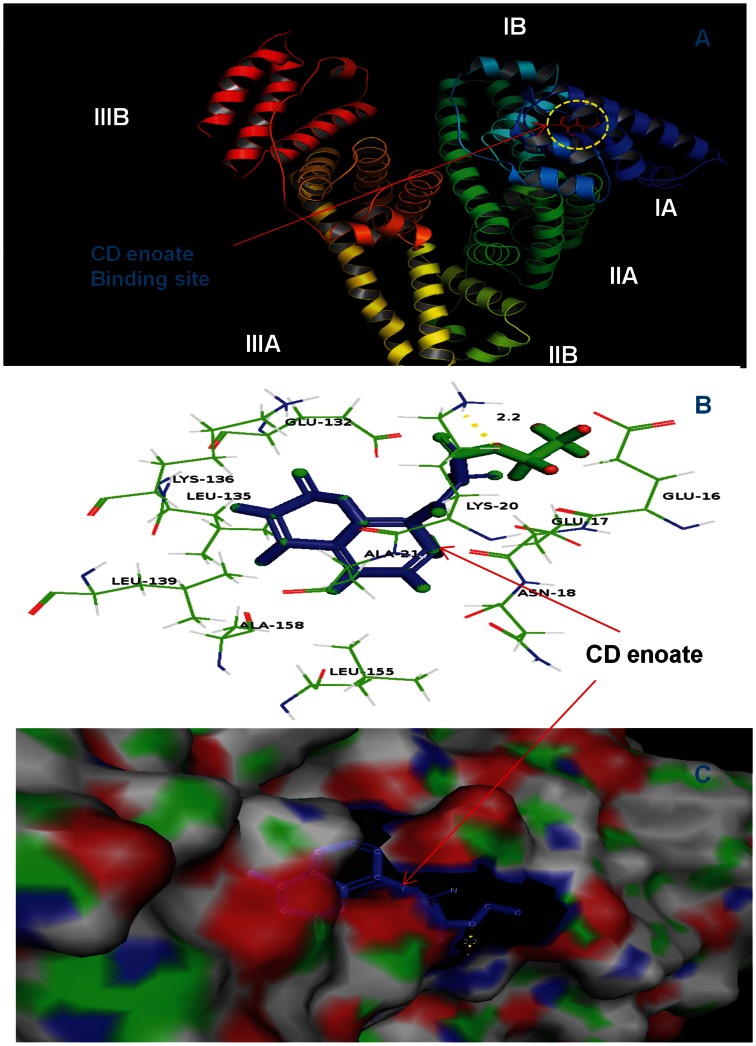
CD enoate docked in the binding pocket of HSA using Autodock4.2. **Different view of HSA and CD enoate docked conformation.** (A) overview in cartoon model of CD enoate binding to HSA, (B) the docking poses of the HSA-CD enoate complex depicted in a ball and stick model (blue), and HSA, represented in line model, (C) hydrophobic pocket of HSA and CD enoate, the CD enoate represents in ball and stick model (green) and the binding pockets in cyanocolor, in which the green color represents the binding pocket residues. Images are generated using PyMol.

**Figure 8:**
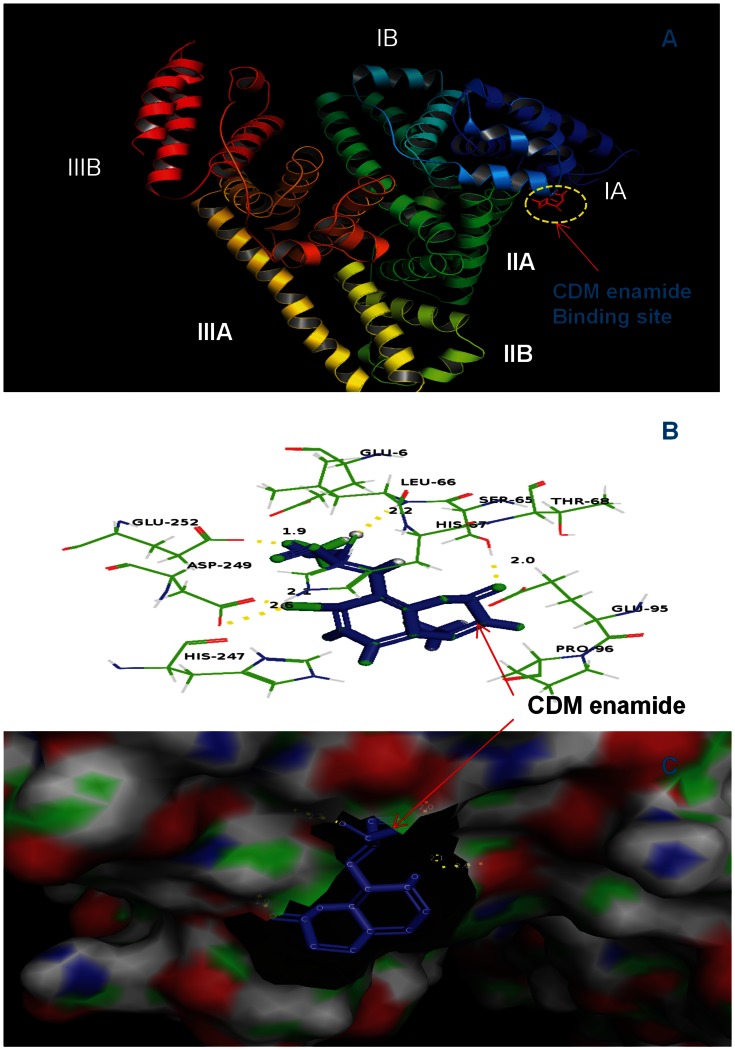
CDM enamide docked in the binding pocket of HSA using Autodock4.2. **Different view of HSA and CDM enamide docked conformation.** (A) overview in cartoon model of CDM enamide binding to HSA, (B) the docking poses of the HSA-CDM enamide complex depicted in a ball and stick model (blue), and HSA, represented in line model, (C) hydrophobic pocket of HSA and CDM enamide, the CDM enamide represents in ball and stick model (blue) and the binding pockets in cyanocolor, in which the green color represents the binding pocket residues. Images are generated using PyMol.

The analysis of crystal structure of HSA has revealed that it consists of a single polypeptide chain of 585 amino acid residues and comprises three structurally homologous domains (I–III): I (residues 1–195), II (196–383), and III (384–585) that assemble to form a heart-shaped molecule [19]. The principal regions of ligand binding sites of HSA are located in hydrophobic cavities in sub-domains IIA and IIIA, which correspond to site I and site II, respectively, other than this there are eight fatty acid binding sites [16], [20]–[23]. Several endogenous compounds and drugs such as bilirubin, hemin, azapropazone, indomethacin, and TIB bind within IB domain as described above [25], [60], [61] including the coumarin derivatives studied here (see Figure 6A, 7A and 8A).

In the present study AutoDock 4.2 program was chosen to examine the binding mode of coumarin derivatives at the active site of HSA. A total of 30 different conformations were generated through blind docking by AutoDock (Table S1). The best score ranked results are shown in Table S1. The docking results showed that CD enamide and CD enoate bind within the binding pocket of subdomain IB whereas CDM enamide bind within the binding pocket of subdomain IB–IIA. The present study indicates that these coumarin derivatives bind mainly to the hydrophobic pocket of subdomain IB (Figure 6C, 7C and 8C). The coumarin derivatives are accommodated in the deepest part of the binding cleft largely shielded from solvent such that the phenyl part is present at the entrance of the pocket and rest of the drug is embedded in the deepest portion of the cleft. The CD enamide, CD enoate, and CDM enamide complexes are stabilized by the hydrogen bond interactions with different bond lengths. The CD enamide complex is stabilized by a hydrogen bond between the drug and Glu 132 amino acid residue of the protein of length 2.0 Å, CD enoate complex by a hydrogen bond between the drug and Lys 20 with the bond length of 2.2 Å and CDM enamide complex by two hydrogen bonds with Ser 65 of lengths 2.0 and 2.2 Å, one hydrogen bond with Glu 252 of length 1.9 Å, and two hydrogen bonds with Asp 249 of lengths 2.1 and 2.6 Å. These molecules specifically interact with different residues located in the sub domain IB, and the complex is stabilized by the hydrogen bonds. The computationally calculated binding energy of lowest energy conformers of CD enamide, CD enoate, and CDM enamide are −6.8 Kcal/mol, −6.69 Kcal/mol and −6.37 Kcal/mol, respectively which are very near to the experimentally measured values. Therefore, the molecular docking and free energy calculation results suggested that coumarin derivatives bound to HSA with both hydrophobic and hydrogen bond interactions.

The moiety of coumarin derivatives was located within the hydrophobic binding pocket and several groups of coumarin derivatives interact with the several residues of sub-domain IB and IIA. Almost all the interactions between the active site and the coumarin derivatives are present within the 4Å moiety [62] which consists of several residues such as LEU17, GLU18, ASN20, LYS21, ALA132, GLU135, LEU136, LYS139, LEU155, LEU158, ALA159, LYS162 and LYS284 for HSA-CD enamide complex, ALA16, GLU17, GLU18, ASN20, LYS21, ALA132, GLU135,LEU136,LYS139, LEU155, LEU158 for HSA-CD enoate complex and GLU6, GLU65, SER66, LEU67, HIS68, THR95, GLU96, PRO247, HIS249 and ASP 252 for HSA-CDM enamide complex (Figure 6B, 7B and 8B).

Therefore, the results of molecular docking indicate that the interaction between coumarin derivatives and HSA are dominated by hydrophobic forces, which is in agreement with the free energy shown by fluorescence data. Our results have shown that coumarin derivatives can interact with HSA within subdomain IB with hydrophobic and hydrogen bonding interactions, which is consistent with the previously published data on other ligand molecules [32]–[34].

### Molecular Dynamics

In order to investigate the stability of the system (protein, ligand, water, ions, etc.) properties were examined by means of rms deviations (rmsd’s) of protein and coumarin derivatives with respect to the initial structure, root mean square fluctuations (RMSF’s) and the radius of gyration (*R*g) of protein. In addition, the stability of system proved the credibility of the docking result, where the coumarin derivatives bound to HSA at subdomain IB were used for MD simulations.

**Figure 9:**
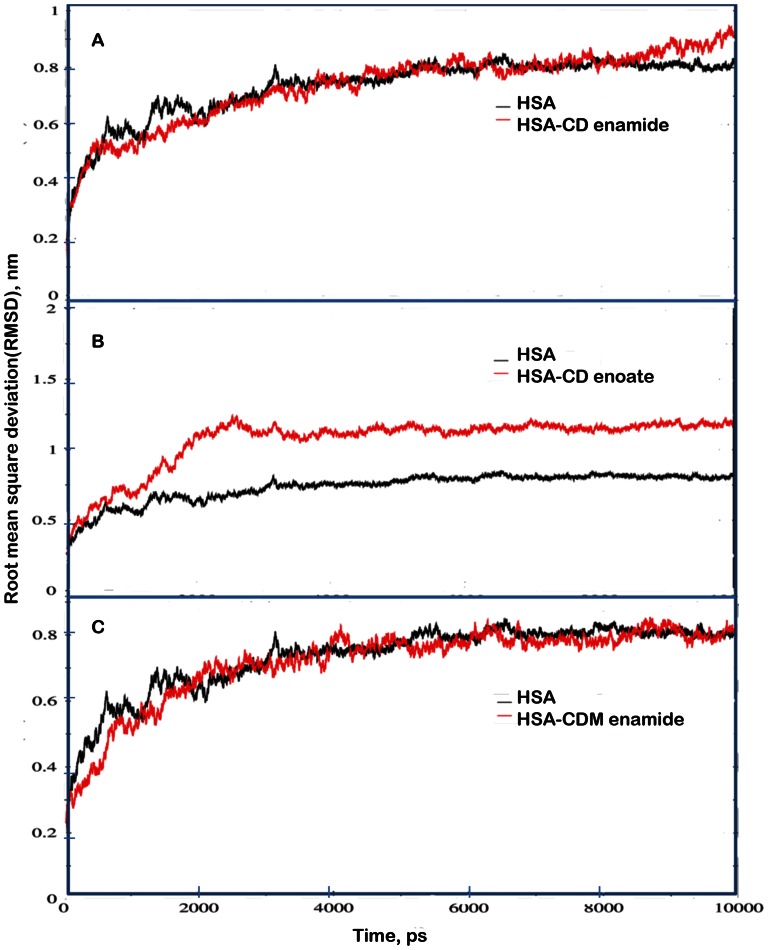
Time dependence of rmsd’s. (A) C_α_ rmsd values for free HSA and HSA-CD enamide complex during 10,000 ps MD simulation. (B) C_α_ rmsd values for free HSA and HSA-CD enoate complex during 10,000 ps MD simulation. (C) C_α_ rmsd values for free HSA and HSA-CDM enamide complex during 10,000 ps MD simulation.

The rmsd values of atoms in pure HSA and HSA-coumarin derivatives complexes were plotted from 0 to 10,000 ps as shown in Figure 9. Analysis of the rmsd values indicates that the rmsd of both systems reaches equilibration and oscillates around in average value after 3000 ps simulation time. The rmsd values of atoms in HSA and HSA-coumarin derivatives complexes were calculated from a 0–10,000 ps trajectory, where the data points were fluctuated for HSA, 0.72±0.036 nm, HSA-CD enamide, 0.85±0.056 nm, HSA-CD enoate, 1.18±0.32 nm, HSA-CDM enamide, 0.82±0.39 nm, respectively. Similar reports were observed from our group that 0.72±0.036 nm, 0.85±0.023 nm, and 0.81±0.032 nm for free HSA, HSA-β-sitosterol and HSA plus betulinic acid, respectively [34], [35].

**Figure 10:**
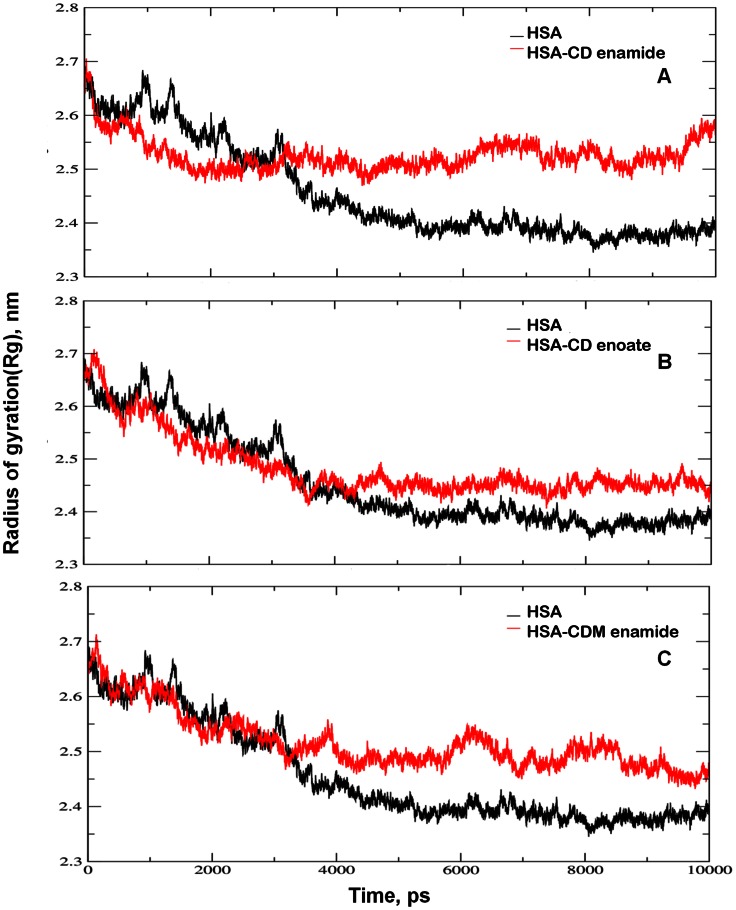
Time evolution of the radius of gyration. (A) radius of gyration (*R*g) values during 10,000 ps of MD simulation of HSA and CD enamide. (B) radius of gyration (*R*g) values during 10,000 ps of MD simulation of HSA and CD enoate. (C) radius of gyration (*R*g) values during 10,000 ps of MD simulation of HSA and CDM enamide.

The integrity of the protein is analyzed by plotting the radius of gyration values against time. Radius of gyration describes the overall spread of the molecule and is defined as the root mean square distance of the collection of atoms from their common centre of gravity. In the present MD studies, we determined the Rg values of free HSA and HSA-coumarin derivatives complexes as shown in Figure 10. The *R*g values were stabilized at about 3500 ps in all the three derivatives of coumarin, indicating that the MD simulation achieved equilibrium after 3500 ps. Initially, the *R*g values of free HSA, HSA-CD enamide, HSA-CD enoate, and HSA-CDM enamide complexes were 2.7 nm. The free HSA, HSA-CD enamide, HSA-CD enoate and HSA-CDM enamide complexes were stabilized at 2.39±0.03, 2.54±0.04, 2.45±0.03, and 2.51±0.04, respectively (Figure 10). Hence, the stabilization of the coumarin-HSA complex is based on the functional groups of the coumarin derivatives.

The previous reports have shown the *R*g value of HSA, determined experimentally from neutron scattering in aqueous solution was 2.74±0.035 nm which indicated that our MD simulations are matching to the experimental values [63]. Also, the present result is in close agreement with our previous report that β-sitosterol and beutilinic acid stabilizes at 2.59±0.03 nm and 2.40+0.031 nm; 2.59±0.03 nm and 2.51±0.01 nm for free HSA and HSA-with complexes, respectively [34], [35]. These results suggest that the radius of gyration value decreased upon binding of coumarin derivatives. This clearly indicates that the coumarin derivatives change the microenvironment of HSA leading to the conformational changes in the HSA during MD simulation. These *R*g values are strongly supported with the experimental CD data that upon binding of coumarin derivatives the protein gets partially unfolded (Figure 4). Thus, our experimental data fully supports the MD simulation in which protein conformational changes occur during binding of coumarin derivatives to HSA. It is clearly shown in MD simulation that HSA-coumarin derivatives complexes were stabilized due to conformational rearrangements. The similar reports were shown by our group that the conformational changes may occur upon binding of ligands during MD simulation [33]–[35].

**Figure 11:**
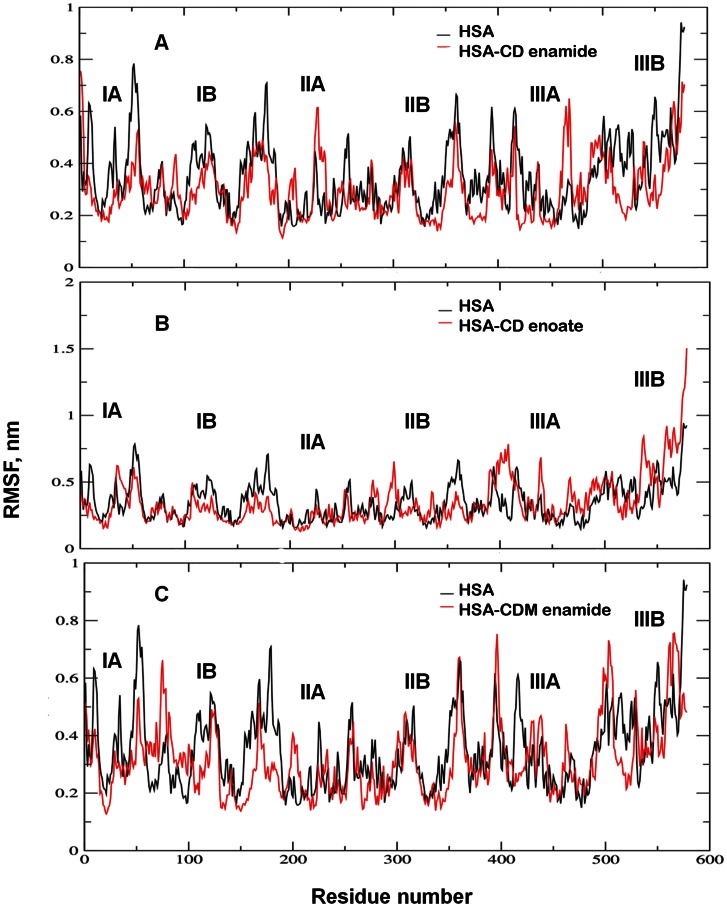
The RMSF values against the residues numbers. (A) the RMSF values of free HSA and HSA-CD enamide complex were plotted against residue numbers. (B) the RMSF values of free HSA and HSA-CD enoate complex were plotted against residue numbers. (C) the RMSF values of free HSA and HSA-CDM enamide complex were plotted against residue numbers.

**Figure 12:**
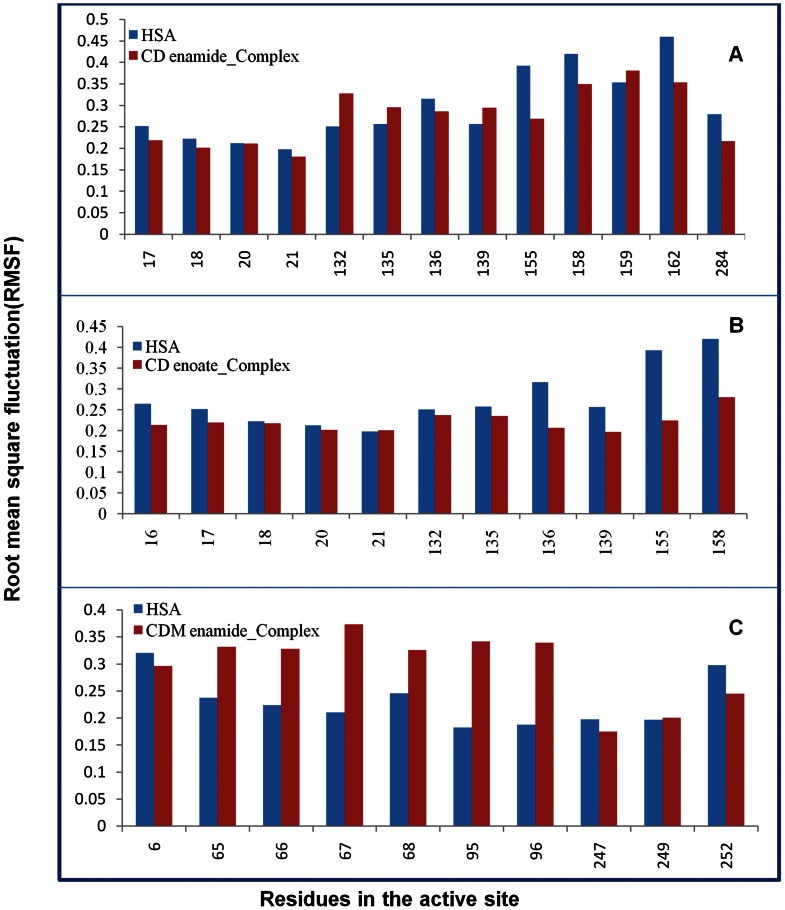
The profile of atomic fluctuations. (A) atomic fluctuations of unliganded HSA and HSA-CD enamide complex (active site amino acid residues present in the IB subdomain of HSA). (B) atomic fluctuations of unliganded HSA and HSA-CD enoate complex (active site amino acid residues present in the IB subdomain of HSA). (C) atomic fluctuations of unliganded HSA and HSA-CDM enamide complex (active site amino acid residues present in the IB-IIA subdomain of HSA).

The fluctuations of a dynamical system (e.g. protein) about some average position or the local protein mobility was analyzed by the time-averaged RMSF values of free HSA and HSA-coumarin derivatives complexes plotted against residue numbers based on the 10,000 ps trajectory data shown in Figure 11. The atomic fluctuations profiles of HSA-coumarin derivatives complexes were found to be very similar to those of HSA. Our results clearly indicate that sub-domain IB in HSA-CD enamide and HSA-CD enoate complexes and subdomains IB and IIA in HSA-CDM enamide complex have lower fluctuations as compared to all other domains. These results suggest that the structure of primary drug binding sites IB and IIA remains rigid during simulation. The loop regions connected to the helices were mainly affected upon binding of coumarin derivatives to HSA. The two loops such as IIa-h2 to IIa-h3 (residues 223–227) and IIIa-h4 to IIIa-h5 (residues 467–470); three loops such as IIa-h6 to IIb-h1 (residues 292–314), IIIa-h3 to IIIa-h4 (residues 436–444) and IIIb-h3 to IIIb-h4 (residues 560–565); three loops such as Ia-h4 to Ia-h5 (residues 76–80), IIIa-h6 to IIIb-h1 (residues 491–510) and IIIb-h3 to IIIb-h4 (residues 560–565) connected to the helices were highly fluctuated upon binding of CD enamide, CD enoate and CDM enamide, respectively to HSA. We investigated the profile of atomic fluctuations of residues CD enamide, CD enoate and CDM enamide in the active site (Figure 12). The fluctuations in the residues in sub-domain IB- E132, L135, K136, L139, L155, A158, K159, K162 in HSA-CD enamide complex; E132, L135, K136, L139, L155, A158 in HSA-CD enoate complex and residues in the subdomain IIA- H247, D249, E252 in HSA-CDM enamide complex are less as compared to other drug binding sites. The Figure 12C clearly shows that in the sub-domain Ia, fluctuations are more, though these are present in the active site as compared to the residues present in the IIa sub-domain. This study provides evidence that HSA binding site IB interacts specifically with CD enamide, CD enoate and IIA with CDM enamide through conformational adjustments of the protein structure, along with the adaptation of ligands conformation to these sites.

### Conclusions

The coumarin derivatives studied here have been shown to have specific binding with HSA. They act as a quencher to absorb fluorescence emitted by HSA at 360 nm. Using Stern-volmer law, the binding constant were determined to be (1.957±0.01)×10^5^, (0.837±0.01)×10^5^ and (0.606±0.01)×10^5^ M^−1^ for 1 molecule of the CD enamide, CD enoate, and CDM enamide binds to HSA, respectively. The standard free energy change were found to be −7.175 Kcal/mol, −6.685 Kcal/mol and −6.49 Kcal/mol for CD enamide, CD enoate and CDM enamide, respectively. The conformational changes were caused by the binding of these molecules to HSA such that the α-helix content decreases with increase in their concentration. Further, molecular docking proves the binding at IB domain and the values of free energy and binding constant coincide for both experimental and computationally obtained values from molecular docking. Results suggested that the formation of hydrogen bond decreased the hydrophilicity and increased the hydrophobicity to stabilize the complexes. Additionally, MD simulation data attributes an important contribution to understanding the effect of the binding of coumarin derivatives on conformational changes of HSA and the stability of a protein drug complex system in aqueous solution. MD simulation studies revealed that HSA and HSA-coumarin derivative complexes reached to equilibration and oscillate around the average value after 3,000 ps and also exhibited conformational change. Later, RMSF curve values for IB domain are almost similar as free HSA indicating binding at IB subdomain. Further, the work gives information about the stability of the HSA-drug complex and this has implication in the pharmacokinetic and pharmacodynamic studies of these molecules. Knowing the precise conditions will greatly help in the study of drug distribution and its delivery. HSA can be assigned a greater role as a carrier molecule for many drugs in the evidence of this work and it would lead to the designing of new inspired coumarin drug molecules. The molecules are chemically synthesized by using novel techniques and success of these drugs shall encourage similar initiatives in the future. Thus, implications range from the pharmaceutical industry to research in chemistry and biology.

## Supporting Information

Figure S1 **Synthesis of coumarin derivatives under Pechmann’s and Knoevengel’s conditions. The compound (I) was treated with N-substituted cyanoacetamide to form** (A) CD enamide. (B) CDM enamide. (C) CD enoate.
(TIF)

Table S1 **Docking Summary of HSA with different coumarin derivatives generated different ligand conformers by the AutoDock program using the Lamarkian Genetic Algorithm.** (1) Docking Summary of HSA with CD enamide. (2) Docking Summary of HSA with CD enoate. (3) Docking Summary of HSA with CDM enamide.
(DOCX)

Table S2 **Secondary structural analysis of HSA and its interaction with** (A) CD enamide. (B) CD enoate. (C) CDM enamide. Based on the Figure 4, the data is analyzed by using CDNN 2.1 software.
(DOCX)
