# Supplementary figures and images for: Elucidation of the Binding Mechanism of Coumarin Derivatives with Human Serum Albumin
Source: PLoS One. 2013 May 28;8(5):e63805. doi: 10.1371/journal.pone.0063805 (PMC3665821; doi:10.1371/journal.pone.0063805)

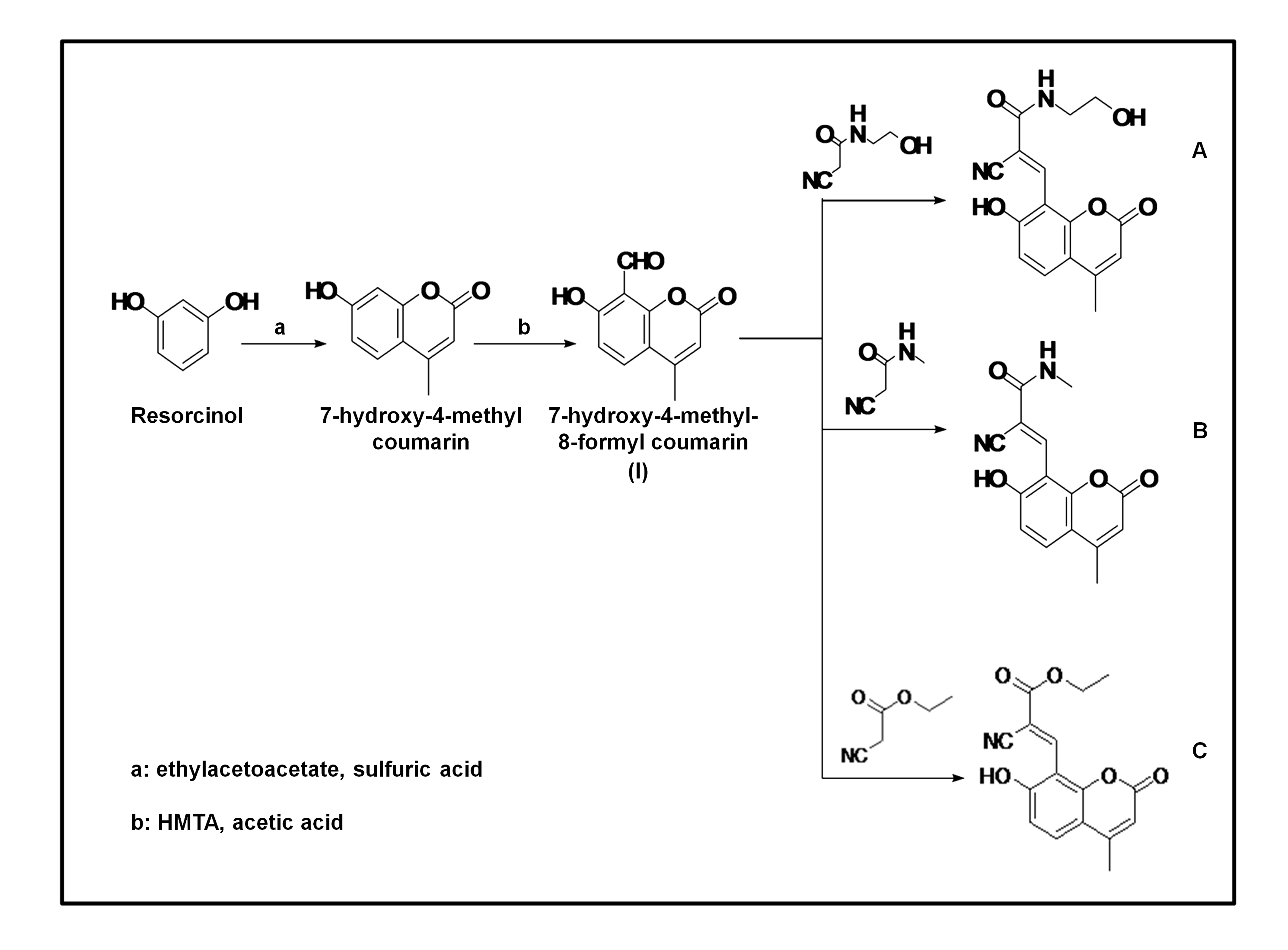

Supplement: Figure S1 — Synthesis of coumarin derivatives under Pechmann’s and Knoevengel’s conditions. The compound (I) was treated with N-substituted cyanoacetamide to form (A) CD enamide. (B) CDM enamide. (C) CD enoate. (TIF) [file pone.0063805.s001.tif]
