# Supplementary material for: Elucidation of the Binding Mechanism of Coumarin Derivatives with Human Serum Albumin
Source: PLoS One. 2013 May 28;8(5):e63805. doi: 10.1371/journal.pone.0063805 (PMC3665821; doi:10.1371/journal.pone.0063805)
Supplement: Table S1 — Docking Summary of HSA with different coumarin derivatives generated different ligand conformers by the AutoDock program using the Lamarkian Genetic Algorithm. (1) Docking Summary of HSA with CD enamide. (2) Docking Summary of HSA with CD enoate. (3) Docking Summary of HSA with CDM enamide. (DOCX) [file pone.0063805.s002.docx]

**Table S1: Docking Summary of HSA with different coumarin derivatives generated different ligand conformers by the AutoDock program using the Lamarkian Genetic Algorithm. (1) Docking Summary of HSA with CD enamide. (2) Docking Summary of HSA with CD enoate. (3) Docking Summary of HSA with CDM enamide.***

1. **CD enamide**

| **Rank** | **Sub-Rank** | **Run** | **Binding Energy [Kcal M^-1^]** | **Inhibitory Constant K_i_** | **K_a_ [M^-1^]** |
| --- | --- | --- | --- | --- | --- |
| **1** | **1** | **7** | **-6.80** | **10.45µM** | **9.56×10^4^** |
| 2 | 1 | 12 | -5.33 | 123.97µM | 8.06×10^3^ |
| 3 | 1 | 11 | -5.22 | 148.09µM | 6.75×10^3^ |
| 4 | 1 | 18 | -5.17 | 161.06µM | 6.21×10^3^ |
| 5 | 1 | 15 | -5.16 | 164.88µM | 6.06×10^3^ |
| 6 | 1 | 4 | -5.09 | 185.00µM | 5.41×10^3^ |
| 7 | 1 | 26 | -5.02 | 209.83µM | 4.76×10^3^ |
| 8 | 1 | 24 | -5.01 | 214.42µM | 4.66×10^3^ |
| 9 | 1 | 5 | -4.73 | 338.25µM | 2.95×10^3^ |
| 10 | 1 | 28 | -4.69 | 362.20µM | 2.76×10^3^ |
| 11 | 1 | 8 | -4.47 | 526.33µM | 1.90×10^3^ |
| 12 | 1 | 25 | -4.36 | 638.33µM | 1.56×10^3^ |
| 12 | 2 | 13 | -4.27 | 737.21µM | 1.36×10^3^ |
| 13 | 1 | 14 | -4.29 | 720.25µM | 1.38×10^3^ |
| 14 | 1 | 17 | -4.19 | 851.30µM | 1.17×10^3^ |
| 15 | 1 | 10 | -4.16 | 897.90µM | 1.11×10^3^ |
| 16 | 1 | 20 | -4.11 | 975.36µM | 1.02×10^3^ |
| 17 | 1 | 9 | -4.10 | 988.98µM | 1.01×10^3^ |
| 18 | 1 | 30 | -4.08 | 1.02mM | 9.80×10^2^ |
| 18 | 2 | 19 | -3.99 | 1.20mM | 8.33×10^2^ |
| 18 | 3 | 16 | -3.91 | 1.37mM | 7.29×10^2^ |
| 19 | 1 | 1 | -3.64 | 2.16mM | 4.63×10^2^ |
| 20 | 1 | 29 | -3.56 | 2.44mM | 4.09×10^2^ |
| 21 | 1 | 22 | -3.51 | 2.69mM | 3.72×10^2^ |
| 22 | 1 | 23 | -3.50 | 2.72mM | 3.67×10^2^ |
| 23 | 1 | 3 | -3.34 | 3.56mM | 2.81×10^2^ |
| 24 | 1 | 2 | -3.32 | 3.70mM | 2.70×10^2^ |
| 25 | 1 | 6 | -2.97 | 6.66mM | 1.50×10^2^ |
| 26 | 1 | 27 | -2.93 | 7.15mM | 1.39×10^2^ |
| 27 | 1 | 21 | -2.35 | 18.95mM | 5.27×10^1^ |

1. **CD enoate**

| **Rank** | **Sub-Rank** | **Run** | **Binding Energy [Kcal M^-1^]** | **Inhibitory Constant K_i_** | **K_a_ [M^-1^]** |
| --- | --- | --- | --- | --- | --- |
| **1** | **1** | **27** | **-6.69** | **12.57µM** | **7.95×10^4^** |
| 2 | 1 | 1 | -5.32 | 125.63µM | 7.96×10^3^ |
| 3 | 1 | 11 | -5.22 | 149.00µM | 6.71×10^3^ |
| 4 | 1 | 9 | -5.04 | 201.91µM | 4.95×10^3^ |
| 5 | 1 | 23 | -5.00 | 216.92µM | 4.61×10^3^ |
| 6 | 1 | 12 | -4.93 | 243.47µM | 4.11×10^3^ |
| 7 | 1 | 22 | -4.91 | 253.39µM | 3.94×10^3^ |
| 8 | 1 | 26 | -4.91 | 253.63µM | 3.94×10^3^ |
| 9 | 1 | 21 | -4.77 | 319.30µM | 3.13×10^3^ |
| 10 | 1 | 4 | -4.76 | 322.68µM | 3.09×10^3^ |
| 10 | 2 | 13 | -4.63 | 404.35µM | 2.47×10^3^ |
| 11 | 1 | 14 | -4.73 | 339.01µM | 2.95×10^3^ |
| 12 | 1 | 19 | -4.67 | 380.55µM | 2.61×10^3^ |
| 12 | 2 | 25 | -4.61 | 414.42µM | 2.41×10^3^ |
| 13 | 1 | 5 | -4.61 | 416.22µM | 2.40×10^3^ |
| 14 | 1 | 10 | -4.56 | 457.52µM | 2.18×10^3^ |
| 15 | 1 | 18 | -4.45 | 548.46µM | 1.82×10^3^ |
| 16 | 1 | 16 | -4.37 | 624.39µM | 1.60×10^3^ |
| 17 | 1 | 20 | -4.24 | 779.65µM | 1.28×10^3^ |
| 18 | 1 | 28 | -4.23 | 799.74µM | 1.25×10^3^ |
| 19 | 1 | 24 | -4.19 | 855.31µM | 1.17×10^3^ |
| 20 | 1 | 8 | -4.11 | 968.47µM | 1.03×10^3^ |
| 21 | 1 | 7 | -4.09 | 999.68µM | 1.00×10^3^ |
| 22 | 1 | 15 | -4.04 | 1.10mM | 9.09×10^2^ |
| 23 | 1 | 6 | -4.04 | 1.20mM | 8.33×10^2^ |
| 24 | 1 | 3 | -3.74 | 1.80mM | 5.56×10^2^ |
| 25 | 1 | 17 | -3.64 | 2.13mM | 4.69×10^2^ |
| 26 | 1 | 29 | -3.59 | 2.34mM | 4.27×10^2^ |
| 27 | 1 | 2 | -3.56 | 2.46mM | 4.06×10^2^ |
| 28 | 1 | 30 | -3.45 | 2.97mM | 3.36×10^2^ |

1. **CDM enamide**

| **Rank** | **Sub-Rank** | **Run** | **Binding Energy [Kcal M^-1^]** | **Inhibitory Constant K_i_** | **K_a_ [M^-1^]** |
| --- | --- | --- | --- | --- | --- |
| **1** | **1** | **5** | **-6.37** | **21.50µM** | **4.65×10^4^** |
| 2 | 1 | 12 | -6.18 | 29.47µM | 3.39×10^4^ |
| 3 | 1 | 2 | -6.15 | 30.91µM | 3.24×10^4^ |
| 4 | 1 | 23 | -5.68 | 68.55µM | 1.46×10^4^ |
| 5 | 1 | 26 | -5.67 | 70.02µM | 1.43×10^4^ |
| 6 | 1 | 8 | -5.54 | 87.48µM | 1.14×10^4^ |
| 6 | 2 | 29 | -5.13 | 172.27µM | 5.80×10^3^ |
| 7 | 1 | 17 | -5.36 | 117.30µM | 8.52×10^3^ |
| 8 | 1 | 18 | -5.33 | 123.86µM | 8.07×10^3^ |
| 9 | 1 | 27 | -5.18 | 160.03µM | 6.25×10^3^ |
| 10 | 1 | 22 | -5.12 | 176.27µM | 5.67×10^3^ |
| 11 | 1 | 30 | -5.12 | 177.73µM | 5.63×10^3^ |
| 12 | 1 | 21 | -5.01 | 214.18µM | 4.67×10^3^ |
| 13 | 1 | 24 | -4.67 | 376.69µM | 2.65×10^3^ |
| 14 | 1 | 11 | -4.67 | 377.37µM | 2.65×10^3^ |
| 15 | 1 | 7 | -4.64 | 395.62µM | 2.53×10^3^ |
| 16 | 1 | 16 | -4.58 | 437.68µM | 2.28×10^3^ |
| 17 | 1 | 28 | -4.57 | 450.04µM | 2.22×10^3^ |
| 18 | 1 | 3 | -4.43 | 567.94µM | 1.76×10^3^ |
| 19 | 1 | 10 | -4.39 | 606.45µM | 1.65×10^3^ |
| 20 | 1 | 15 | -4.36 | 642.03µM | 1.56×10^3^ |
| 21 | 1 | 13 | -4.18 | 861.64µM | 1.16×10^3^ |
| 22 | 1 | 4 | -4.17 | 872.25µM | 1.15×10^3^ |
| 23 | 1 | 20 | -4.12 | 961.87µM | 1.04×10^3^ |
| 24 | 1 | 1 | -4.03 | 1.11mM | 9.01×10^2^ |
| 25 | 1 | 14 | -3.93 | 1.32mM | 7.57×10^2^ |
| 26 | 1 | 6 | -3.80 | 1.64mM | 6.09×10^2^ |
| 27 | 1 | 9 | -3.68 | 2.02mM | 4.95×10^2^ |
| 28 | 1 | 25 | -3.37 | 3.36mM | 2.98×10^2^ |
| 29 | 1 | 19 | -2.75 | 9.71mM | 1.03×10^2^ |

* The lowest free energy conformations are shown by the bold font which are the most stable conformations matching with the fluorescence spectrometry experiments.
