# Supplementary material for: Elucidation of the Binding Mechanism of Coumarin Derivatives with Human Serum Albumin
Source: PLoS One. 2013 May 28;8(5):e63805. doi: 10.1371/journal.pone.0063805 (PMC3665821; doi:10.1371/journal.pone.0063805)
Supplement: Table S2 — Secondary structural analysis of HSA and its interaction with (A) CD enamide. (B) CD enoate. (C) CDM enamide. Based on the Figure 4, the data is analyzed by using CDNN 2.1 software. (DOCX) [file pone.0063805.s003.docx]

**Table S2: Secondary structural analysis of HSA and its interaction with (A) CD enamide. (B) CD enoate. (C) CDM enamide. Based on the Figure 4, the data is analyzed by using CDNN 2.1 software.**

|  | **HSA** | **HSA+0.001mM Drug** | **HSA+0.003 mM Drug** | **HSA+0.005 mM Drug** |
| --- | --- | --- | --- | --- |
| Helix(%) | 57.3±1.2 | 54.5±1.14 | 53.10±1.11 | 47.10±0.98 |
| Antiparallel(%)  **A** | 6.05±1.1 | 6.08±1.04 | 6.5±1.18 | 8.0±1.45 |
| Parallel(%) | 6.65±0.4 | 7.45±0.44 | 7.7±0.46 | 8.7±0.52 |
| Beta-Turn(%) | 12.2±0.9 | 13.7±1.01 | 13.8±1.01 | 14.7±1.08 |
| Rndm. Coil(%) | 17.8±1.6 | 18.1±1.62 | 18.9±1.69 | 21.5±1.93 |
| Helix (%) | 57.3±1.3 | 56.8±1.26 | 53.9±1.19 | 52.6±1.16 |
| Antiparallel(%) | 6.05±1.1 | 6.1±1.07 | 6.3±1.14 | 7.0±1.27 |
| Parallel(%)  **B** | 6.65±0.4 | 7.1±0.42 | 7.5±0.45 | 8.0±0.48 |
| Beta-Turn (%) | 12.2±0.9 | 13.3±0.98 | 13.7±1.01 | 13.9±1.02 |
| Rndm. Coil (%) | 17.8±1.7 | 18.0±1.8 | 18.6±1.77 | 18.5±1.76 |
| Helix (%) | 57.3±1.6 | 57.0±1.6 | 55.8±1.56 | 42.5±1.2 |
| Antiparallel(%)  **C** | 6.05±0.7 | 6.15±0.71 | 6.35±0.7 | 8.8±1.0 |
| Parallel (%) | 6.65±0.0 | 6.8±0.0 | 7.15±0.0 | 9.2±0.0 |
| Beta-Turn (%) | 12.2±0.9 | 13.1±0.96 | 13.5±0.99 | 15.2±1.12 |
| Rndm. Coil (%) | 17.8±1.8 | 18.1±1.83 | 18.7±1.9 | 24.3±2.45 |
